# Pubic Lice (*Pthirus pubis*): History, Biology and Treatment *vs*. Knowledge and Beliefs of US College Students

**DOI:** 10.3390/ijerph6020592

**Published:** 2009-02-06

**Authors:** Alice L. Anderson, Elizabeth Chaney

**Affiliations:** Carol Belk Building, Department of Health Education and Promotion, Environmental Health Program, East Carolina University, Greenville, NC, 27858, USA; E-Mail: chaneye@ecu.edu (E. C.)

**Keywords:** *Pthirus pubis*, pubic lice, college student, survey

## Abstract

Pubic lice (*Pthirus pubis*) maintain a worldwide parasitic population infesting two to over 10 percent of human populations, continuing a presence that has been constant since early evidence 10,000 years ago. Outbreaks in the 1970s have been recorded, but incomplete records preclude description of a definitive population cycle. Current levels of infestation in a US college student population were investigated in this study. Knowledge and opinions of students were also recorded in an online survey administered to college students taking a basic health course at a mid-sized East Coast University. In a group of 817 students, 35 reported experience with pubic lice or other STD infection. Knowledge, beliefs, and treatment attitudes were examined for the 782 students who did not have experience with either pubic lice or STD infection. These students deemed antibiotics as a viable treatment for pubic lice infestation. They also indicated negative attitudes toward the use of pesticide crèmes, which are the most useful prescription. Symptoms and transmission myths in student answers are described.

## 1. Introduction

Pubic lice infestations are not a reportable condition in the US, but are considered an STD. There have been reports of outbreaks of pubic lice during earlier decades [1], though reports are not numerous. In countries where military or STD records are widely available, records are more common. Mimouni [2], for example, describes two outbreaks found in Israeli army records during the years 1972–1999. Incidence of pubic lice in European and South American STD clinic records and prison health records have been reported sporadically [3–5]. Though reporting on pubic lice is less frequent than head lice or body lice, its epidemiology is important because of correlation with the occurrence of other STD infections [2,6–8]. Because there is evidence of co-occurrence with other STD infections, and because accurate surveillance of ectoparasite levels is valuable in evaluating emerging or changing trends, this study was undertaken to describe self-reported attitudes and behavior concerning lice prevention and treatment (and treatment myths) which will add to the general knowledge of *Pthirus pubis* infestation in young adults. It will also help alert college health clinics and health education providers to the need for continued educational efforts about this pest.

### 1.1. Pubic lice Biology and History

Before discussing current student attitudes and health behaviors, a general history of pubic louse occurrence and biology will facilitate evaluating the scope of student understanding There is archeological and historical evidence of lice infestation in the human population for as long as 10,000 years [9], though there is much less occurrence information for pubic lice than for head and body lice. Archaeological evidence for occurrence of lice is unusual, due in part to burial and preservation methods which called for cleaning bodies and removing body parasites before burial [10]. Kenward [10], however, reports finding evidence of pubic lice in Roman and Medieval Britain. In South America, Rick, *et al.* [11], report evidence of pubic lice in human remains. Reinhart and Buikstra [12] report that these archeological studies confirm the parasitological axiom that “10% of the population harbors 70% of the parasites”. Thus, pubic lice have been present in the human population for thousands of years, but have never been of major importance as a serious pest.

**Figure 1.**
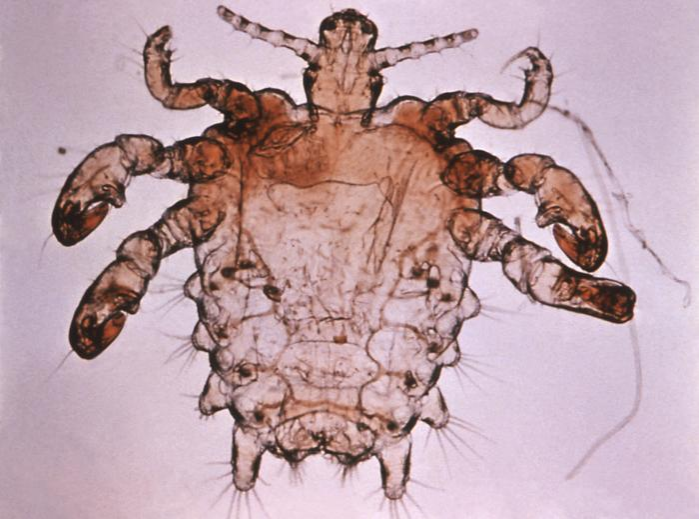
Photograph of pubic louse (CDC).

The biological evolution of pubic lice and head or body lice shows divergence morphologically into two distinct species of ectoparasites. Together, the human louse complex comprises two species: *Pediculus humanus* (variants *Pediculus humanus* var. *corporis* and *Pediculus humanus* var. *capitis* are the first. Pubic lice are a second distinct genus and species, *Pthirus pubis* (Figure 1). Head lice (*Pediculus humanus* var. *capitus* are the most commonly occurring of the three species, particularly in school children. Body lice are the only disease vector of the complex, capable of transmitting bacterial diseases including trench fever, epidemic typhus, and louse-borne relapsing fever [13]. Unlike head lice and pubic lice, body lice are associated typically with poor hygiene, socioeconomic status and disasters [14].

Pubic lice are adapted to a sedentary life style on pubic hair, and sometimes on eyelashes and body hair, not often leaving the infested body. They are usually transmitted during sexual contact, and have been associated with other sexually transmitted diseases [2,6,7,8]. All lice infestations are diagnosed by identification of live adult lice, and viable eggs (nits) on the hair shafts in the specific body regions giving them their names [15]. Empty egg cases attached to hair shafts are not diagnostic of an active infection. Treatment for lice infestations is summarized by the following table derived from Diaz [14], and Leone [16] (Table 1).

As noted in Table 1, resistance to the pesticides in pediculocide treatments is increasing. Only pyrethrins+PBO pesticide formulations are available in the US.

### 1.2. Pubic Lice Incidence in Recent Surveys

Typical *Pthirus pubis* infestation burden in the world appears to be approximately 2% of the (mainly) adult population. Records are often related to STD clinic records or to travel data. In a recent report regarding numbers and types of ectoparasites in travelers returning to the UK, only 7 out of 73 (about 1.6%/year) insect specimens collected from symptomatic patients during the years 1994 to 2000 were identified as *Pthirus pubis* [14]. In countries where travelers may have visited, however, infestations numbers were considerably higher. In Nepal, for example, a control group for a study of lice in children showed pubic lice prevalence at 7% for pubic lice with head lice, and 9% for pubic lice with body lice [17]. Bignell [8] found 3.5 % male and 2% female infestation with pubic lice in genitourinary clinic screening in the UK in 1991, but only 1% in 2004. Varela, *et al.* [3] found a yearly infestation rate in Spanish STD clinic records of 1.3 to 4.6% over the years 1988 to 2001. In Australia, Hart [18] reported that from 1988–1991 the incidence of *Pthirus pubis* in men attending an STD clinic was 1.7% and in women 1.1%. Other articles from 1990–2006 included pubic louse infestation in STD clinic screening and prostitute screening of 2.2% infestation [5].

In addition to maintaining information about infestation rates for pubic lice, it is also important to determine the level of information susceptible populations may have concerning treatment and transmission. Information about possible co-occurring STD infections and about effective treatment are needed for susceptible populations, such as college students, where sexual activity typically ranges from 70–90% [19]. This project, therefore, was undertaken to survey college students at a medium-sized East Coast University regarding student experience, knowledge and attitudes about pubic lice and infestation prevention and treatment. Cultural myths, prejudice, stigma and shame may prevent individuals from seeking treatment for pubic lice and possibly other STD infections [9,20], which in turn prevents accurate surveillance, and can confound appropriate health intervention measures.

## 2. Results and Discussion

**Figure 2.**
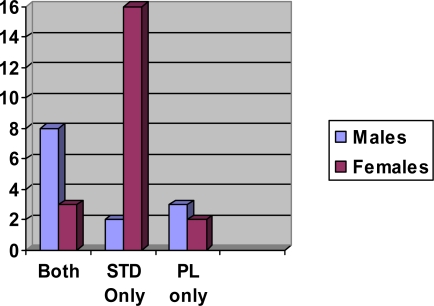
Numbers of college students with experience of pubic lice, STD infection or both by sex.

Table 2 is the list of questions administered to 817 college students regarding pubic lice. The results in the table include answers of 782 students who self-reported no experience with pubic lice or STDs. The survey population comprised both male and female students. Ninety-five percent were ages 17–23. Male students made up 35% of the respondents, and female students 65%. Eleven out of 817 students who answered all 26 pubic lice questions had self-reported experience with pubic lice infestations (1.346%). Thirty-two out of 817 students had self-reported experience with STD infection (4%). Some of these had both STD and pubic lice experience and a few only pubic lice experience. Male students reported more experience with pubic lice and females with other STD infection (Figure 2).

Attitudes about environmental treatment for pubic lice included mostly positive responses to all suggested actions. For actions involving pesticides, however, negative responses increased (Table 2). Attitudes regarding transmission of pubic lice elicited generally positive answers for all of the possibilities listed, including using a toilet seat after someone who was infested. Negative answers were highest for living with but not sleeping with a person who was infested. Responses to the questions listing possible symptoms resulted in positive responses for all descriptions, including symptoms of swollen genitals, and vaginal or penile discharge (Table 2). The two symptoms with the most positive responses were itching in affected areas and evidence of lice eggs on pubic hair.

Self-treatment questions including bathing in Lysol or bleach water were answered positively by some students. Use of hydrocortisone on bites and taking of antibiotics were the two self-treatments, in addition to discontinuing intimate contact, were answered positively most often.

Low levels of pubic lice incidence (nearly 2%) in a sexually active population is within the expectation of a group of young adults as indicated in recent literature of STD clinic surveys. Attitudes and knowledge about treatment and symptoms suffer from persistent myths and misinformation, however. Some students recorded a negative reaction to pesticides as a treatment for ectoparasites. This eliminates the only effective treatment for killing lice other than mechanical removal. Use of shampoos or crèmes containing pyrethrin pesticides is recommended to prevent the spread of these ectoparasites [9]. Physical removal of all insects and eggs is difficult to accomplish, but would be effective if completely thorough.

A second source of myth and possible stigma is peer group misinformation about pubic lice behavior and transmission. Students regarded environmental contact with toilet seats and clothing after an infested person as dangerous. Since pubic lice are extremely sedentary and seldom leave close contact with the body, transmission through either objects or clothing is highly unlikely [8]. Close intimate contact or “skin to skin” contact is the main source of transmission.

Symptom misconceptions appear to be the source of most confusion. Since these students have little experience with pubic lice infestations, non-specific symptoms such as fever and a generalized rash were considered viable, though itching and evidence of lice eggs were decisive favorites and legitimate diagnostic symptoms.

Answers to treatment option questions showed negative attitudes toward an effective treatment with pesticide creme. The positive choice of antibiotic treatment, an ineffective treatment, interestingly exceeded even the choice of fever as a symptom, which might warrant the use of antibiotics (80% chose antibiotic use and 32% chose fever as a symptom). Discontinuation of contact with current sexual partner was the overall choice action for prevention.

Limitations of this study included the use of student populations of different ages in college classes as the study group. The older, returning adult students, skewed the average age of students who had experience with STD infection. The small number of students with self-reported experience of pubic lice or STD infection prevented generalization or characterization of students with experience. Self-reported data often includes inaccurate or dishonest answers, which affects incidence characterization and generalizations. Extrapolation of the results of this study to the general public thus may be limited since the subjects were all students who volunteered to answer the survey. To ensure IRB standards, students were given the option to omit some of the questions, and surveys with omitted questions were not included in the final tabulations Some bias in the results may have occurred as a result of this; however, the incomplete questionnaires were not a systematically related group according to the overall demographic data which all students did complete.

## 3. Experimental Section

An online survey was administered to college students taking a basic health course at a mid-sized East Coast University to determine their knowledge, experience and attitudes about pubic lice infestations, prevention and treatment. Students volunteered to complete the survey, and also had the option to omit any questions they did not wish to answer in the survey if they chose to participate. Incomplete surveys were not counted in the analysis. Access to the results of the survey was restricted to faculty members who obtained University IRB review for the questions (UMCIRB#07-0590), and who were trained in IRB methodology. Only students in the class had access to the survey through the campus server. Students who chose to answer the survey as part of their class work were issued a receipt to print, with the time and date of completion recorded. Completion was verified by instructors in each of the student’s class sections. Results without individual identification were sent to researchers through university computer servers.

## 4. Conclusions

In an article surveying stigma and shame related to STD infection, Lichtenstein [21] reported that pubic lice were the least stigma and shame eliciting STD among seven others, including HIV, syphilis, gonorrhea, genital warts, genital herpes, and Chlamydia. It is possible that since the advent of HIV as a fearful world-wide scourge, pubic lice and other STD infections have lost significance, importance or stigma in the list of consequences from risky sexual behavior. Ectoparasitic population dynamics should still be a concern of health scientists, however, in this current climate of worldwide human population homogenization from travel and collaborative work. In an increasingly homogenous world, *Pthirus pubis* has a potential for new outbreak emergence, especially with its increased resistance to pesticides. Its cousin, *Pediculus humanus humanus* is a vector of disease, so pubic lice and other lice population and infestation statistics cannot safely be ignored. Pubic lice especially warrant attention and continued inclusion in health education for young adults because of their relation to STD infection, and their classification as a sexually transmitted disease. Serious attitude and knowledge misconceptions such as the overwhelming approval of antibiotic use for treatment of pubic lice need to be addressed.

## Figures and Tables

**Table 1.** Recommended pediculocide treatments for pediculosis, revised in 2007 [14,16].

Treatment	Safety profile/use	Efficacy	Resistance of insect to treatment
0.33% pyrethrins + PBO shampoo	Excellent Apply to hair, wash off after 10 min.	95% ovicidal in Susceptible strains	Increasing
1% to 5% permethrin cream rinse	Excellent 1% creme rinse, wash off after 10 min.	2 week residual	Increasing
0.5% malathion lotion shampoo **(not available in US)**	Flammable, organophosphate poisoning risks.	95% ovicidal in susceptible strains, rapid killing, good residual	Increasing
1% lindane lotion and shampoo **(not recommended in the US)**	Potential CNS toxicity from organochlorine poisoning. Only use as last resort Wash off after 4 min.	95% ovicidal, no residual	Increasing
Ivermectin 0.8 % shampoo **(not available in US)**	Excellent Apply to hair, wash off after 5 min.	Excellent	None

**Table 2.** Survey Questions and responses: College student knowledge and beliefs about pubic lice.

*Question*	*Yes*	*No*	*N/A*
**Which of the following would you use to treat the environment if you had pubic lice?**			
1. Buy new bedding	690	92	0
2. Wash clothing	759	19	4
3. Wash bed linens	760	17	5
4. Spray clothing with insecticide for lice	659	116	7
5. Spray bed linens with insecticide for lice	674	106	2
6. Other treatment	716	66	0
7. No special treatment of the environment is required	86	689	7
**Can pubic lice be transmitted from one person to another through...**			
8. Shared clothing with a person who has them?	735	44	3
9. Skin to skin contact with the affected area of a person who has them?	724	54	4
10. Generally sharing a living space but not sleeping with someone who has them?	549	231	2
11. Using a toilet seat after someone who has them?	582	198	2
**Which of the following are symptoms of pubic lice (crab lice)?**			
12. Pink rash all over body	307	472	4
13. Itching in affected areas	763	15	4
14. Tiny purplish spots in the affected area	598	199	4
15. Fever	254	523	5
16. Swollen genitals	524	255	3
17. Discharge (fluid) from the vagina or penis	387	392	3
18. Visualizing lice in the pubic hair	658	120	4
19. Evidence of lice eggs on pubic hair	702	76	4
**If you become infected with pubic lice, what actions should you take in addition to seeking assistance from a health care provider?**			
20. Bathe in Lysol or bleach water	213	566	3
21. Use hydrocortisone on bites	634	143	5
22. Take antibiotics	623	152	7
23. Use pesticide containing creme	485	290	7
24. Discontinue contact with current intimate partner, and inform them of lice	731	45	6
25. Have you ever had pubic lice (crab lice)?	16	760	6
26. Have you ever had any other sexually transmitted disease?	32	750	1
